# Involvement of Auxin Biosynthesis and Transport in the Antheridium and Prothalli Formation in *Lygodium japonicum*

**DOI:** 10.3390/plants10122709

**Published:** 2021-12-09

**Authors:** Natsumi Ohishi, Nanami Hoshika, Mizuho Takeda, Kyomi Shibata, Hisakazu Yamane, Takao Yokota, Masashi Asahina

**Affiliations:** 1 Graduate School of Science and Engineering, Teikyo University, 1-1 Toyosatodai, Utsunomiya 320-8551, Tochigi, Japan; moon.gaia@gmail.com (N.O.); hyamane7179@gmail.com (H.Y.); yokota@nasu.bio.teikyo-u.ac.jp (T.Y.); 2 Department of Biosciences, Teikyo University, 1-1 Toyosatodai, Utsunomiya 320-8551, Tochigi, Japan; onion.star11@gmail.com (N.H.); 1016b4@gmail.com (M.T.); kyomi@nasu.bio.teikyo-u.ac.jp (K.S.); 3 Advanced Instrumental Analysis Center, Teikyo University, 1-1 Toyosatodai, Utsunomiya 320-8551, Tochigi, Japan

**Keywords:** antheridium, auxin, gibberellin, abscisic acid, *Lygodium japonicum*, protonema

## Abstract

The spores of *Lygodium japonicum*, cultured in the dark, form a filamentous structure called protonema. Earlier studies have shown that gibberellin (GA) induces protonema elongation, along with antheridium formation, on the protonema. In this study, we have performed detailed morphological analyses to investigate the roles of multiple phytohormones in antheridium formation, protonema elongation, and prothallus formation in *L. japonicum*. GA_4_ methyl ester is a potent GA that stimulates both protonema elongation and antheridium formation. We found that these effects were inhibited by simultaneous application of abscisic acid (ABA). On the other hand, IAA (indole-3-acetic acid) promoted protonema elongation but reduced antheridium formation, while these effects were partially recovered by transferring to an IAA-free medium. An auxin biosynthesis inhibitor, PPBo (4-phenoxyphenylboronic acid), and a transport inhibitor, TIBA (2,3,5-triiodobenzoic acid), both inhibited protonema elongation and antheridium formation. *L. japonicum* prothalli are induced from germinating spores under continuous white light. Such development was negatively affected by PPBo, which induced smaller-sized prothalli, and TIBA, which induced aberrantly shaped prothalli. The evidence suggests that the crosstalk between these plant hormones might regulate protonema elongation and antheridium formation in *L. japonicum*. Furthermore, the possible involvement of auxin in the prothalli development of *L. japonicum* is suggested.

## 1. Introduction

Ferns constitute the most diverse group of vascular plants, after seed plants, and are thought to have appeared on Earth more than 300 million years ago [1,2]. Ferns evolved after the diversion of bryophytes, including hornworts, liverworts, and mosses, which constitute the three early-diverged clades of land plants [3]. During this evolutionary process, bryophytes acquired simple reproductive ability by separating the sexes, while seed plants evolved with sophisticated reproductive ability by developing floral organs. Because ferns are evolutionally situated between bryophytes and seed plants, investigation of fern reproduction might provide a springboard for clarifying the evolution of reproductive mechanisms.

Prothalli, the gametophyte stage of ferns, are developing antheridium and archegonium that produce sperm and eggs, respectively. Upon fertilization, sporophytes begin to emerge, leading to spore production. In bracken fern (*Pteridium aquilinum*), meristic gametophytes with organized meristem develop into a heart-shaped gametophyte, while non-meristic gametophytes are irregularly shaped and form only antheridia [4]. The Japanese Climbing Fern, *Lygodium japonicum,* develops prothalli under natural light conditions, where the archegonium forms around the notch and the antheridium around the rhizoid [5]. *L. japonicum*, and some other ferns, are known to secrete pheromone-like substances called antheridiogens (antheridium inducers), including GAs that control the ratio of males to females and induce the formation of antheridium to ensure genetic diversity in the population [6,7,8].

In *L. japonicum*, rapidly growing meristematic gametes form archegonium, while others form antheridium within the germinating population. Moreover, it has been found that sensitivity to GA varies with the size of individuals [9]. GA is accepted by a receptor called GID1 (GA-insensitive dwarf 1); when GID1 receives a GA molecule, the DELLA protein, an inhibitor of gibberellin response, is degraded [10,11]. It has been reported that the expression level of gibberellin biosynthetic genes is increased in mature *L. japonicum* prothalli and that the expression level of GID1 is increased in immature prothalli [9]. These studies hypothesized that archegonia formed in mature individuals secrete GAs, which accelerate the development of antheridia in the surrounding young individuals, leading to the production of sperm to be accepted by archegonia. 

**Figure 1:**
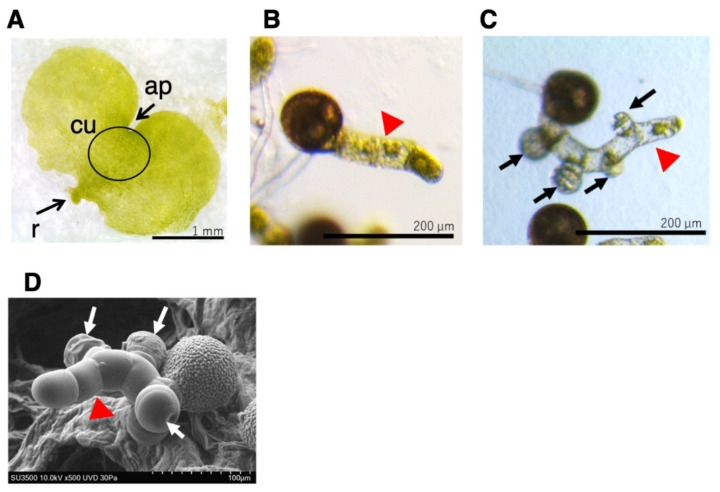
Prothalli, germinating protonema, and protonema with antheridia. (**A**) Germinating spore cultured in MS medium for approximately 20 days under white light formed prothalli. Scale bar indicates 1 mm. ap, apical notch; cu, cushion; r, rhizoid (**B**) Germinating spore cultured in MS medium containing 10–100 nM GA_4_-Me in the dark for 3 days (**B**) or 7 days (**C**) after red light irradiation. The scale bars indicate 200 µm. (**D**) SEM image of germinating spore cultured in MS medium containing GA_4_-Me in the dark for approximately 10 days after red light irradiation. The scale bar indicates 100 µm. Arrows (black and white) indicate antheridium and red arrowheads indicate protonema.

When grown in a medium containing GA under red light irradiation, *L. japonicum* spores are germinated, and as the resulting protonemata elongates, antheridia are formed on them (Figure 1). The antheridium primordium is formed from the mitotic plane, obliquely introduced at the upper end of the cells near the spore shell of the protonema [4], and comprises three jacket cells (basal cells, ring cells, and apical cap cells) that surround 16 or 32 sperm cells. Protonema consists of a single row of cells, and due to its morphological simplicity, has been used as a powerful tool to search for antheridium inducers of ferns [12,13,14,15]. Such experimental techniques are also utilized in this study.

However, very little is known about whether plant hormones other than GAs are involved in this antheridium formation process, and how they interact with each other. IAA occurs in various species of plants, and is known to induce cell division, elongation, and differentiation, leading to fruit growth, apical and lateral bud development, as well as stem and root elongation, which have been investigated at the cellular and molecular levels. In addition, the biosynthesis of IAA from tryptophan has been shown to proceed through multiple pathways, among which a two-step reaction catalyzed by tryptophan aminotransferase (TAA) and flavin-containing monooxygenase (YUCCA) is a common feature in land plants [16,17]. In *Zea mays,* transcriptome analysis has demonstrated that genes encoding auxin-responsive transcription factors ARFs and TIR1 act as receptors for auxin. Even in *Marchantia polymorpha*, these genes are expressed during phloem formation and female reproductive organ formation [18]. Furthermore, in *Physcomitrella patens*, auxin-responsive genes and the PIN protein, responsible for auxin polarity transport, have been expressed during oogenesis and antheridium development [19]. Auxin-mediated signaling pathways for adventitious root development have been found to be conserved in Arabidopsis and the fern *Ceratopteris richardi* [20], suggesting that ferns also should maintain similar genetic diversity of seed plants, with respects to reproductive organ formation. However, auxin biosynthesis, as well as physiological functions, such as antheridium formation in ferns, have not yet been investigated in detail. Hickok and Kiriluk [21] reported that gametophyte formation in the fern *Ceratopteris thalictroides* was inhibited by exogenous auxins. Furthermore, the normal male and female ratio caused by an antheridiogen was significantly increased by IBA (indole-3-butyric acid). However, this effect was deemed to be indirectly elicited by IBA-mediated growth inhibition, and hence it was assumed that IBA did not directly substitute for antheridiogen. In this study, we have investigated how plant hormones GA, IAA, and ABA, which are known to be endogenous [12], are mutually interrelated in protonema elongation and antheridium formation in *L. japonicum*.

## 2. Results and Discussion

### 2.1. Effect of ABA and IAA on GA_4_-Me-Medaited Protonema Elongation and Antheridium Formation of L. japonicum

*L. japonicum* protonema is a filamentous tissue (Figure 1B), and GA_4_-Me induces single or multiple antheridia on it as spherical cells (Figure 1C), which can be observed under light and electron microscopes. The average length of protonema cultured in the medium containing 100 nM GA_4_-Me was 146.6 ± 21.7 µm (N = 60), while the antheridia were formed on almost all of the measured protonemata (Figure S1). The average number of antheridia on individual protonema was 2.05 ± 0.49 (N = 60). 

**Figure 2:**
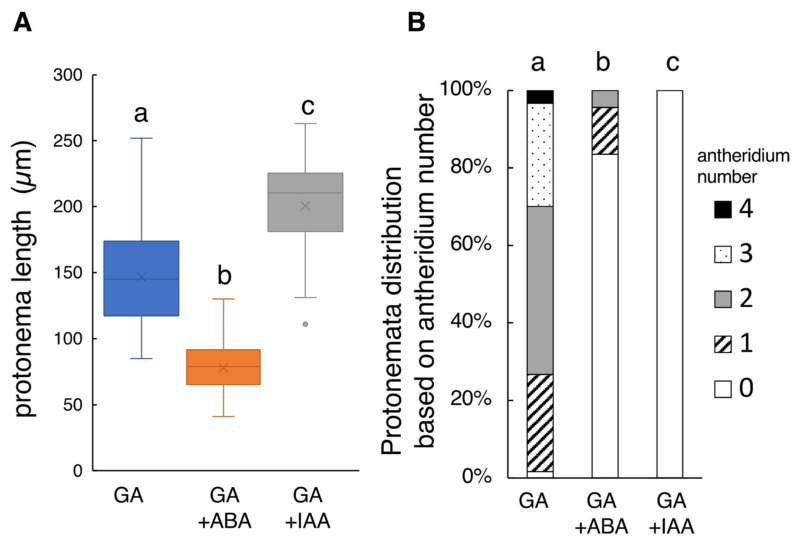
Effect of simultaneous application of GA_4_-Me and ABA or IAA treatment on protonema elongation and antheridia formation. (**A**) Boxplot for protonema length in each treatment. The bars indicate maximum and minimum values, and interquartile ranges were shown. X indicates the mean value. (**B**) Protonemata distribution based on antheridium number. The average number of antheridia per individual spore in GA alone, GA + ABA, and GA + IAA were 2.05 ± 0.49, 0.23 ± 0.31, and 0, respectively. Statistical analysis was performed using the Steel–Dwass test. Different letters indicate statistically significant differences (*p* < 0.05; N = 60 for GA alone; N = 76 for GA + ABA; N = 44 for GA + IAA).

Because Yamane et al. [22] reported that ABA did not significantly inhibit the germination of *L. japonicum* spores in the range of 1 µM to 0.1 mM ABA under white light condition, 10 µM ABA was added to the culture medium to see any effects on the protonema growth. As a result, 10 µM ABA drastically altered the tissue morphology (Figure S1), with the protonema length being reduced to 78.02 ± 10.79 µm (N = 76) (Figure 2A), approximately 50% of the control (GA_4_-Me alone). The number of protonema-carrying antheridia also greatly decreased to approximately one fifth (0.23 ± 0.31 antheridia, *p* < 0.05; Figure 2B).

This finding indicates that ABA may be concerned with protonema elongation and antheridia formation. However, the fact that antheridia were observed even in some of the protonema whose elongation was restricted seems to suggest that antheridia formation is not directly suppressed by ABA. Hõrak et al. [23] reported that ABA is involved in stomatal opening and closing of *Dryopteris crassirhizoma,* while McAdam et al. [24] demonstrated that SnRK2, which is involved in ABA signaling of Arabidopsis, regulates sexual differentiation by suppressing spermatogenesis in the hypocotyl in *Ceratopteris richardii.* Therefore, the functions and signaling pathways of ABA in ferns might be similar to those in higher plants. However, the physiological function and molecular mechanism of ABA in fern were not fully elucidated to date. 

In the presence of GA, protonema cells elongated in a straight fashion with transverse divisions. Simultaneous application of 10 µM IAA induced abnormal swelling due to longitudinal cell division, and no apparent antheridium was observed (Figure S1). The mean length of the protonema was 200.52 ± 19.21 µm (N = 44) (Figure 2B), approximately 1.3 times longer than the control (Figure 2B). These findings suggest that such abnormal morphology of protonema cells should be responsible for the antheridium deficiency. 

Altogether, it may be assumed that the signaling and biosynthesis of IAA, and also ABA, are prerequisite factors in antheridium formation in *L. japonicum.*

### 2.2. Effect of Transferring of IAA-Treated Cells to an IAA-Free Medium

We examined whether the inhibitory effects of IAA were restored by transferring it to IAA-free media. Spores were cultured on GA plus IAA medium for 5 days, and the germinated spores were transferred to a newly prepared GA medium and hormone-free medium. As a reference experiment, the germinated spores were transferred to GA plus IAA medium. The protonema length of the cells transferred to GA medium and hormone-free medium was reduced, compared to that of the control (Figure S2). Antheridium formation was only slightly recovered by transferring to a hormone-free medium, but was significantly rescued by GA medium (Figure S2). However, the recovered antheridium was not formed on cells lined up in series, but on sites where the cells had divided into two or three rows. This finding suggests that such abnormal protonema morphology is not directly linked to disrupted antheridium formation. It may be conceivable that normal cell division occurred again in the IAA-free medium, and then antheridium was formed from the newly formed cells. These results indicated that GA was required for the formation of antheridium and antheridia were induced from certain cells after protonema elongation, and that auxin promotes cell division while inhibiting antheridium formation induced by GA.

### 2.3. Effect of Auxin Inhibitors on Protonema Elongation and Antheridium Formation

The evidence cited above suggests that auxin is involved in both protonema elongation and antheridium formation. Therefore, we investigated whether this effect was suppressed by IAA biosynthesis inhibitors, such as PPBo and kynurenine. PPBo is an analog of yucasin, which strongly inhibits auxin synthesis by interacting with the YUCCA protein family [25,26]. Kynurenine is an auxin biosynthesis inhibitor that competitively inhibits TAA1/TAR activity. Simultaneous application of PPBo and Yucasin to *Zea mays* was found to inhibit IAA biosynthesis in an additive manner [27].

**Figure 3:**
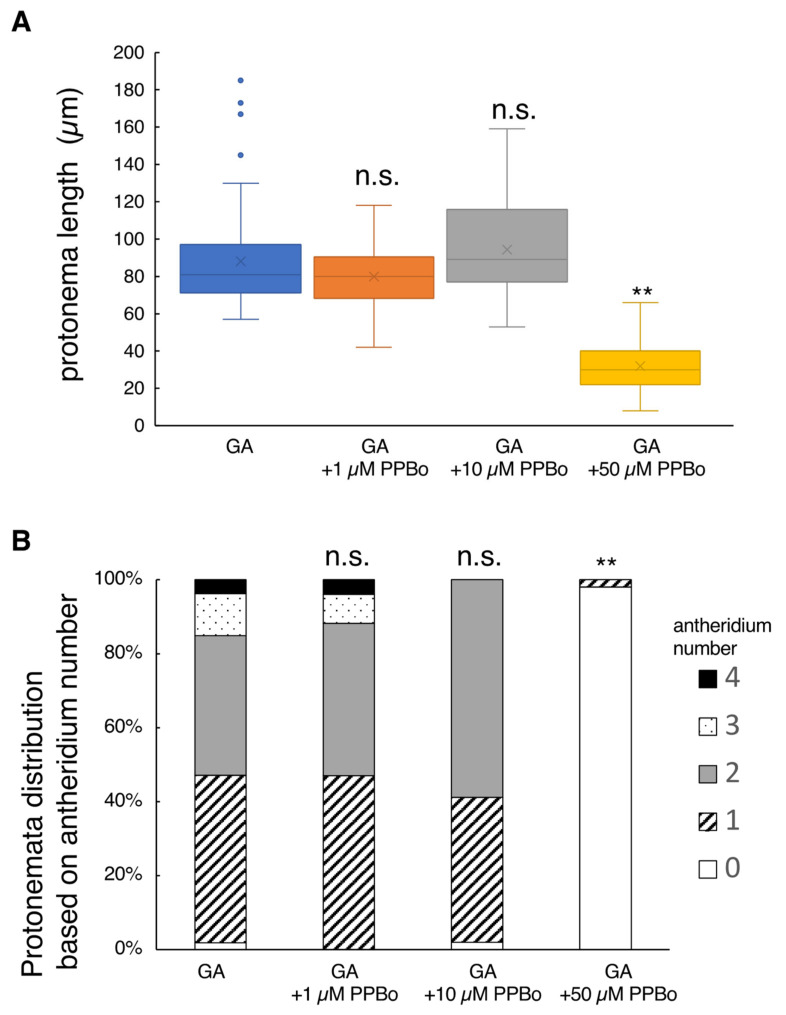
Effect of IAA biosynthesis inhibitor on protonema elongation and antheridia formation. Germinating spores were cultured with 10 nM GA_4_-Me alone or with simultaneous application of PPBo at 1, 10, and 50 µM (N = 51). All cells were cultured in the dark for 7 days after red light irradiation. (**A**) Boxplot for protonema length in each treatment. The bars indicate maximum and minimum values, and interquartile ranges were shown. X indicates the mean value. Asterisks indicate statistically significant differences compared with GA alone (Steel’s test; n.s., not significant; **, *p* < 0.01). (**B**) Protonemata distribution based on antheridium number. The average number of antheridia per individual spore in GA alone or with 1, 10, and 50 µM PPBo were 1.6 ± 0.1, 1.7 ± 0.11, 1.6 ± 0.08, and 0.02 ± 0.02, respectively. Asterisks indicate statistically significant differences compared with GA alone (Steel’s test; n.s., not significant; **, *p* < 0.01).

The addition of 1 or 10 µM PPBo to the basal growth media containing GA showed no effects on *L. japonicum* tissues, but the application of a high concentration of 50 µM PPBo suppressed protonema elongation to a large extent where antheridium formation was almost completely suppressed (Figure 3). Application of 10 µM yucasin alone or 10 µM kynurenine alone did not significantly affect either the protonema length or antheridium development compared to controls (GA alone) (Figure S3). The application of a mixture of yucasin and kynurenine partially inhibited antheridium formation, but did not affect protonema elongation (Figure S3).

**Figure 4:**
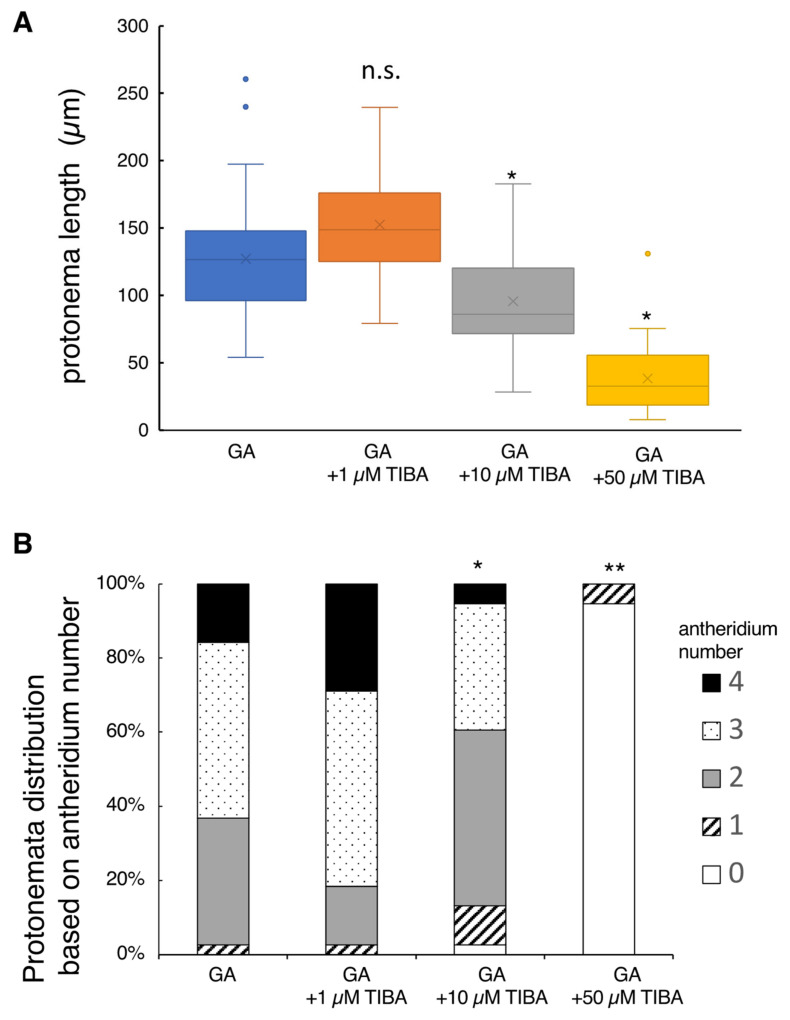
Effect of IAA transport inhibitor on protonema elongation and antheridium formation. Germinating spores were cultured with 100 nM GA_4_-Me alone or with simultaneous application of TIBA at 1, 10, and 50 µM. (**A**) Boxplot for protonema length in each treatment. The bars indicate maximum and minimum values, and interquartile ranges were shown. X indicates the mean value. Asterisks indicate statistically significant differences compared with GA alone (Steel’s test; n.s., not significant; *, *p* < 0.05). (**B**) Protonemata distribution based on antheridium number. The average number of antheridia per individual spore in GA alone or with 1, 10, and 50 µM TIBA were 2.76 ± 0.43, 3.08 ± 0.43, 2.29 ± 0.48 and 0.05 ± 0.13, respectively. Asterisks indicate statistically significant differences compared with GA alone (Steel’s test; *, *p* < 0.05; **, *p* < 0.01). N = 38.

Adding 1 µM TIBA (auxin polar transport inhibitor) to the basal culture medium containing GA did not significantly affect protonema elongation (Figure 4). However, the application of 10 µM or 50 µM TIBA reduced the protonema length to 80% and 30%, respectively, when compared to the controls (GA alone). Antheridium formation profiles observed in the 1 µM and 10 µM TIBA treatments were similar to those of the controls (Figure 4). In contrast, the application of 50 µM TIBA severely inhibited antheridium formation; this inhibition is likely the secondary toxic effect because 95% of spores did not germinate at this concentration. It should be noted that the spore germination rates observed in 1 µM TIBA, 10 µM TIBA, and control (GA alone) media ranged from 30% to 40%, but in 50 µM TIBA, it drastically reduced to 5% (Table S1).

Previous transcriptome analyses assumed that YUCCA proteins, which are IAA biosynthesis enzymes, as well as PIN proteins, which are polar transporters of IAA, occur in the protonema of *L. japonicum* [28]. Therefore, it is presumed that *L. japonicum* has mechanisms for auxin biosynthesis and polar transport, similar to higher plants. PPBo treatment suppressed protonema elongation, but unexpectedly suppressed antheridium formation significantly at the same time (Figure 3). Furthermore, yucasin, kynurenine, and their combination did not affect protonema elongation (Figure S3). This inconsistency may be due to the auxin-synthetic enzymes of *L. japonicum*, which have different specificities from those of seed plants. However, the inhibition of protonema elongation by TIBA supports our hypothesis (Figure 4). Altogether, it seems that IAA is at least partially involved in the elongation of protonema in *L. japonicum*.

In seed plants, GA and auxin both regulate cell elongation and tissue differentiation. These hormones are intimately involved in many light-regulated developmental processes, such as hypocotyl elongation, shade avoidance, and photomorphogenesis [29,30,31]. Auxin is also known to affect GA signaling and biosynthesis [29,30,32].

In the *P. patens*, it has been suggested that auxin biosynthesis, homeostasis, and responsiveness appear important for the final differentiation of the egg and upper basal cells [19], and also that the differentiation from chloronemata to caulonemata is regulated by auxin and light signaling [33]. An auxin antagonist that was specific for the IR1/AFB auxin receptor (BH-IAA) completely inhibited auxin-induced caulonemata differentiation. These results suggested that auxin and light signals are integrated to regulate protonema differentiation in *P. patens* [34]. Furthermore, inhibition of *ent*-kaurene production in *P. patens* conferred auxin tolerance in protonema development and affected photomorphogenesis in protonema. Previous studies reported that the biologically active GAs present in angiosperms were not detected in the *P. patens*, suggesting that GA precursors including *ent*-kaurene themselves have biological roles similar to those of GAs that accelerate fern spore germination [35,36]. Hayashi et al. [34] reported that terpene growth regulators interact with other hormones such as auxin and light signals to control developmental processes, including caulonema differentiation. Altogether, it seems most plausible that interactions of auxin with GA and light signaling are required for the morphogenesis and sexual organ differentiation in *L. japonicum.* In addition to GA, IAA, and ABA, brassinosteroids (BRs) have recently been shown to occur in *L. japonicum* tissue [37]. Synergisms of IAA with BRs occur in many systems [38]. Therefore, the interaction of IAA with BRs also may be a future research target.

### 2.4. Effects of Auxin Inhibitors on the Morphology of L. japonicum Prothalli

**Figure 5:**
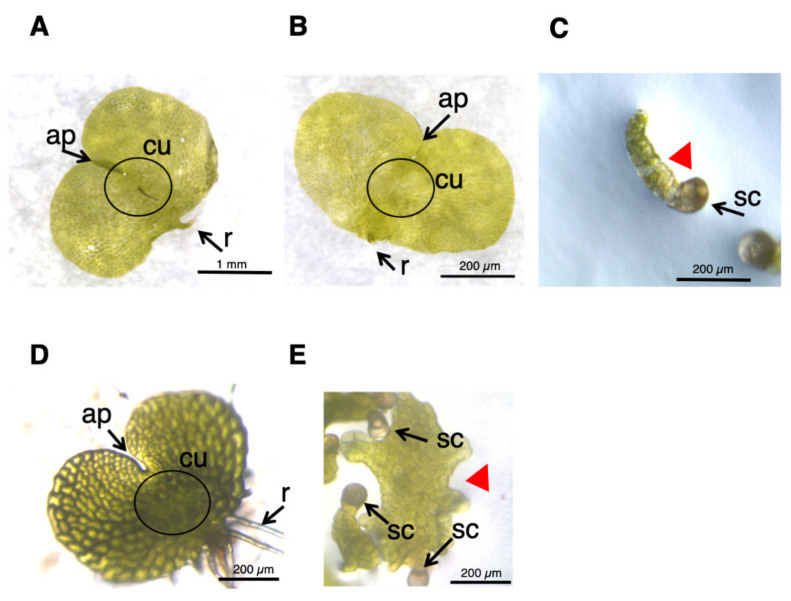
Effect of IAA inhibitors on prothalli formation. (**A**–**C**) Germinating spores cultured in MS medium (**A**) and MS medium containing 50 µM yucasin (**B**) or 50 µM PPBo (**C**) under white light for approximately 40 days. Scale is 1 mm (**A**), 200 µm (**B**), or 200 µm (**C**), respectively. (**D**,**E**) Germinating spores cultured in MS medium (**D**) and MS medium containing 10 µM TIBA (**E**) for approximately 20 days under white light. The scale is 200 µm. Representative results are shown. ap, apical notch; cu, cushion; r, rhizoid. sc, spore coat. Red arrowheads indicate aberrantly shaped prothalli.

*L. japonicum* spores were germinated in GA medium under white light, and ca. one month later, the cultures produced normal prothalli, as shown in Figure 5A. When 50 µM yucasin was supplied to the medium (Figure 5B), the prothalli obtained were indistinguishable from those of the normal cells (Figure 5A). On the other hand, the tissue grown in 50 μM PPBo medium remained at the stage of germinating spores, and no development of prothalli was observed (Figure 5C), suggesting that high-concentration PPBo caused IAA deficiency, restricting two-dimensional cell expansion. Application of auxin biosynthesis inhibitor inhibited the formation of prothalli in the light and produced a morphology similar to that in the dark. These results suggested that auxin also has an important role in photomorphogenesis in *L. japonicum.* To confirm whether auxin is involved in prothalli morphogenesis under light conditions, we also performed an analysis using TIBA. When germinating spores were grown in a medium containing 10 µM TIBA for about 20 days, irregularly shaped prothalli, rather than normal heart-shaped prothalli, were produced (Figure 5D,E), indicating that auxin transport is important in prothalli development. These results suggest that the morphogenesis of the prothalli of *L. japonicum* might be regulated through mutual interactions between GA and auxin.

Among known plant hormones, auxin is the most influential, in that it plays multifaceted roles in many plants, including morphogenesis and embryogenesis. Recently, Aya et al. [28] constructed a transcriptome database of *L. japonicum* and identified genes homologous to known auxin-responsive genes, auxin biosynthesis genes, and auxin transporter genes. Such information is essential for creating transformants, which, in turn, should become a prerequisite for detailed genetic and molecular analyses. However, regrettably, no effective techniques have so far been available to make transformants of *L. japonicum*, such as auxin reporter genes DR5::GUS or DR5::GFP, as well as gene knockout mutants and overexpressing transformants. These will hopefully become future tools to understand the roles of auxin in ferns in depth.

## 3. Materials and Methods

### 3.1. Plant Materials and Culture Conditions

Spores of *Lygodium japonicum* (Thumb.) Sw. were sterilized in sodium hypochlorite solution (6% effective chlorine concentration) for 5 min, centrifuged at 3000 rpm for 30 s, and then washed with sterile distilled water. This process was repeated twice, and then the spores were suspended in sterile D.W. Murashige and Skoog (MS) medium [39] with 0.5% (*w*/*v*) agar adjusted to pH 5.5 was used for the cultivation of *L. japonicum* spores. Plant hormones and their inhibitors used in this study were GA_4_-Me, ABA, IAA, 5-[4-chlorophenyl]-2,4-dihydro-[1,2,4]-triazole-3-thione (Yucasin), 4-phenoxyphenylboronic acid (Kynurenine), 4-phenoxyphenylboronic acid (PPBo) and 2,3,5-triiodobenzoic acid (TIBA). These chemicals were dissolved in MeOH (GA, ABA, and IAA) or DMSO (Kynurenine, PPBo, and TIBA) and pipetted into 12-well plates (MS-BC12R, Sumitomo Bakelite, Tokyo, Japan), and the solvent (MeOH) was air-dried aseptically before 2 mL MS agar medium was dispensed. The final concentrations of hormones were adjusted to 10 or 100 nM (GA_4_-Me) or 100 µM (ABA and IAA), and kynurenine, PPBo, and TIBA were adjusted to 1, 10, or 50 µM (0.1% DMSO). PPBo and kynurenine were kindly gifted by Prof. T. Koshiba (Tokyo Metropolitan University) and Prof. K. Hayashi (Okayama University of Science), respectively.

After solidification of the medium, approximately two drops of sterilized *L. japonicum* spore suspension were dispensed into each hole with a Pasteur pipette. The plates were kept in the dark in an incubator (25 °C; Nippon Medical & Chemical Instruments Co., Osaka, Japan) for 2–5 days and then irradiated with red light for 24 h to induce germination. The plates were then incubated in the dark for an additional seven days.

### 3.2. Measurement of Protonema Elongation and Antheridium Formation

Protonemata and antheridia were fixed in 2.5% glutaraldehyde (0.2 M phosphate buffer, pH 7.4) and observed under a stereomicroscope (M165FC, Leica Microsystems, Wetzlar, Germany). The length of the protonema and the number of antheridia were measured using ImageJ (https://imagej.nih.gov/ij/ (accessed on 1 November 2021)). The numbers of antheridia were only counted as morphologically distinct individuals. Experiments were performed using at least three independent plant samples. Scanning electron microscopy (SEM) observations were performed using SU3500 (Hitachi-Hitech Co., Tokyo, Japan) after ionic liquid coating (HILEM IL1000, Hitachi-Hitech) at an acceleration voltage of 10 kV.

### 3.3. Effect of Auxin Inhibitors on Prothalli Development

Plates were prepared using the same procedure as in the protonema culture and then incubated in an incubator in the dark for 2–5 days. After spore germination, the plates were further incubated under white light at 25 °C for up to 40 days before being observed under a stereomicroscope.

## 4. Conclusions

We examined the effects of endogenous plant hormones, auxin and ABA, on protonema elongation and antheridium formation induced by GA in *L. japonicum.* It was concluded that the morphogenesis of the reproductive organs and prothalli of the fern might be regulated through the interaction of auxin and ABA with GA. 

## Data Availability

The data presented in this study are available in the article.

## Supplementary Materials

The following are available online at https://www.mdpi.com/article/10.3390/plants10122709/s1, Figure S1: Effect of simultaneous application of GA_4_-Me and ABA or IAA treatment on protonema elongation and antheridium formation. Protonema cultured with 100 µM GA_4_-Me alone or simultaneous application of ABA or IAA. Arrows indicates antheridium and red arrowheads indicate protonema. The scale bars indicate 200 µm; Figure S2: Growth medium replacement experiments. (**A**) Boxplot for protonema length in each treatment. The bars indicate maximum and minimum values, and interquartile ranges were shown. X indicates the mean value. (**B**) Protonemata distribution based on antheridium number. Different letters indicate statistically significant differences (Steel–Dwass test, *p* < 0.05); Figure S3: Effect of IAA biosynthesis inhibitor treatment on protonema elongation. (**A**) Boxplot for protonema length in each treatment. The bars indicate maximum and minimum values, and interquartile ranges were shown. X indicates the mean value. n.s., not significant. (**B**) Protonemata distribution based on antheridium number. Asterisks indicate statistically significant differences compared with GA alone (Steel’s test; n.s., not significant; *, *p* < 0.05); Table S1: Germination rate on medium containing GA_4_-Me with TIBA.
