# Supplementary material for: Involvement of Auxin Biosynthesis and Transport in the Antheridium and Prothalli Formation in Lygodium japonicum
Source: Plants (Basel). 2021 Dec 9;10(12):2709. doi: 10.3390/plants10122709 (PMC8706445; doi:10.3390/plants10122709)
Supplement: Supplementary file 1 [file plants-10-02709-s001.zip › plants-1472175-supplementary.pdf]

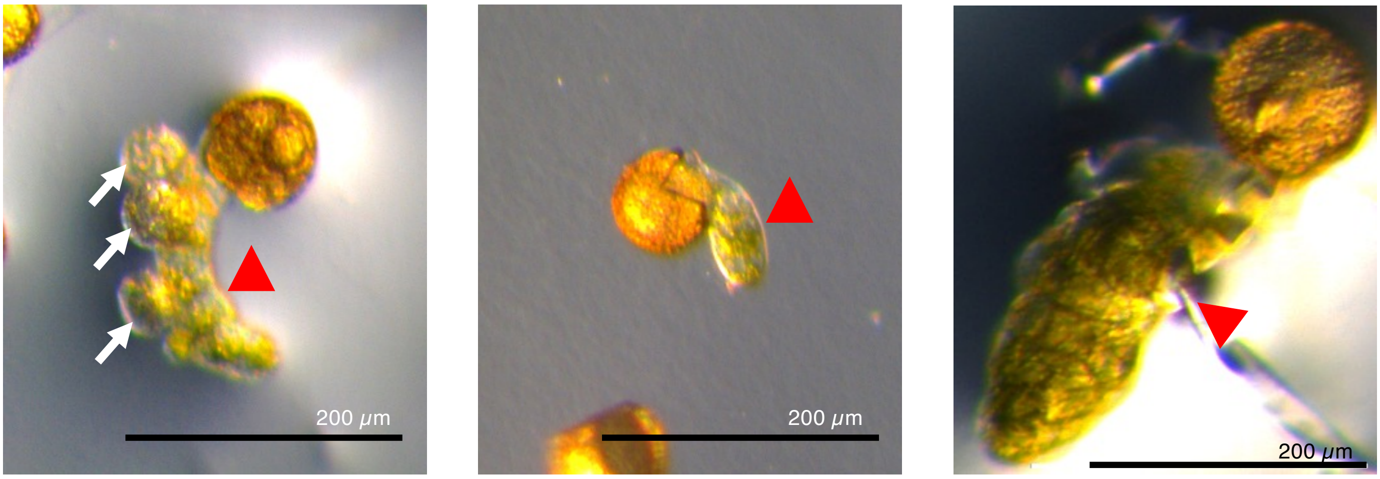

GA GA+ABA GA+IAA

Figure S1

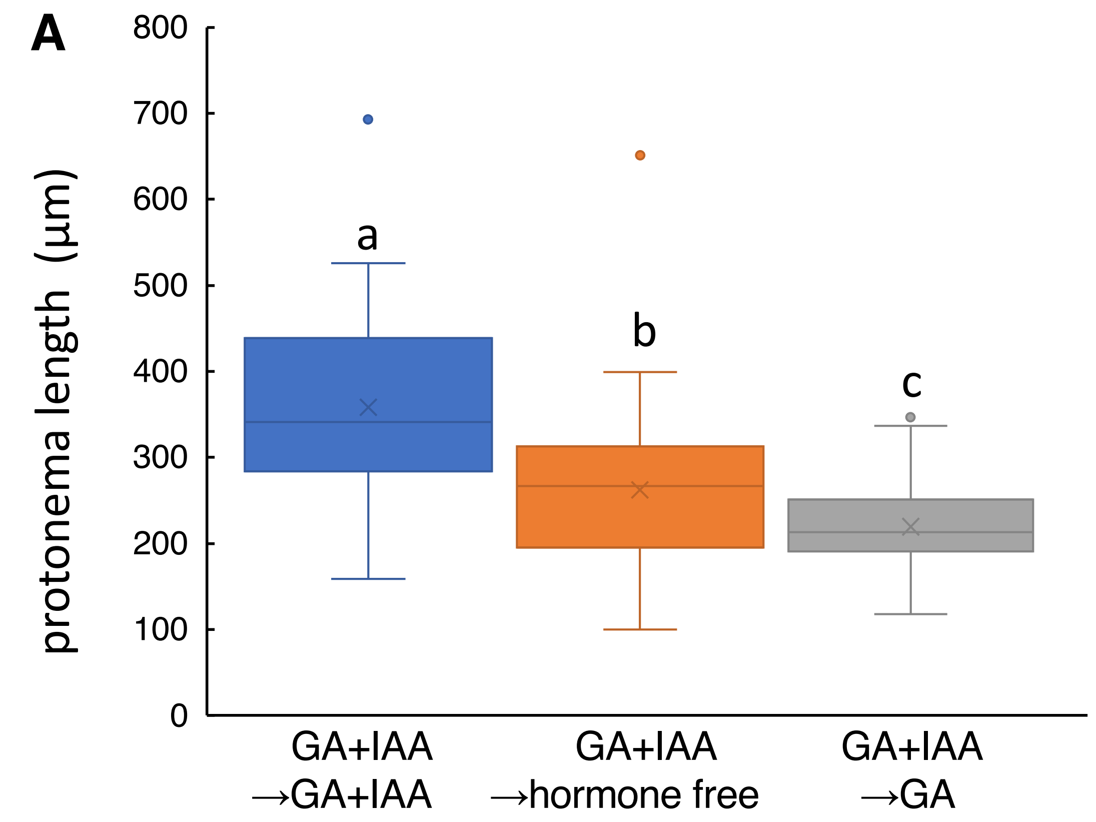
A
protonema length (μm)
800
700
600
500
400
300
200
100
0
a
b
c
GA+IAA →GA+IAA
GA+IAA →hormone free
GA+IAA →GA

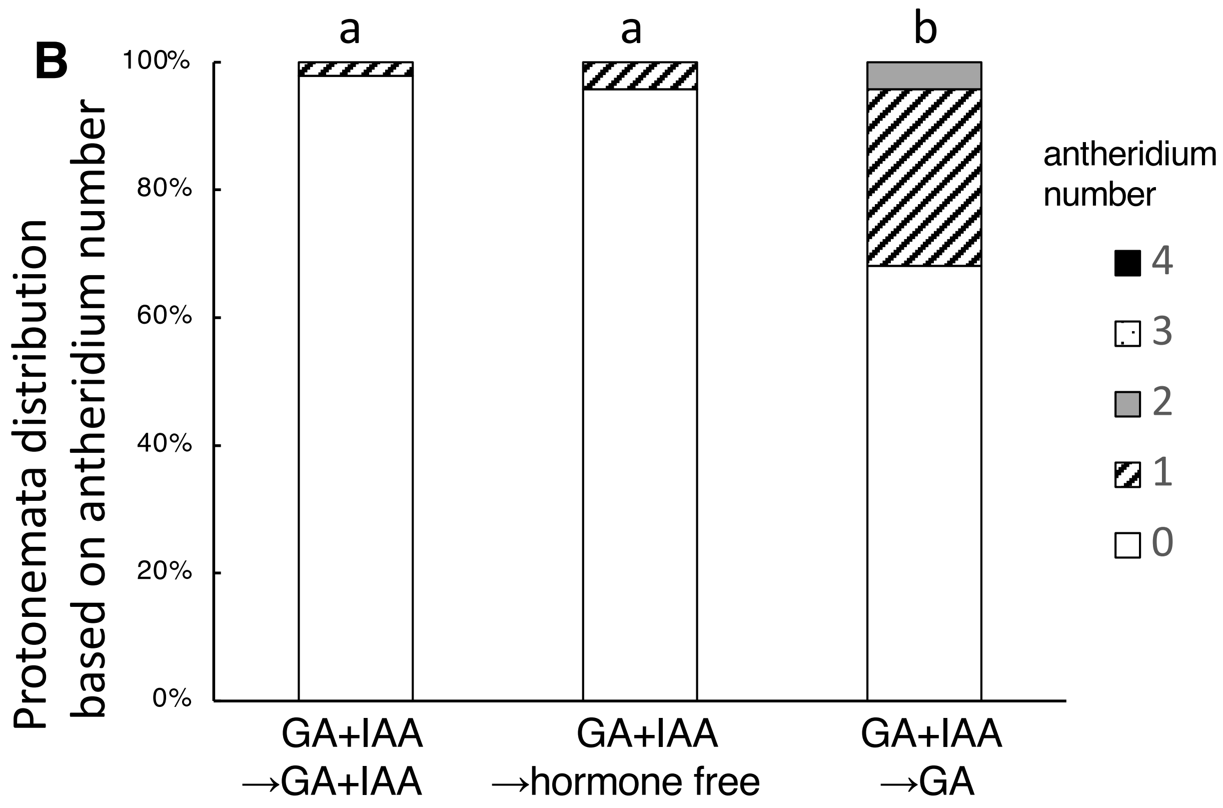
B
Protonemata distribution based on antheridium number
100%
80%
60%
40%
20%
0%
a
a
b
GA+IAA →GA+IAA
GA+IAA →hormone free
GA+IAA →GA
antheridium number
4
3
2
1
0

Figure S2

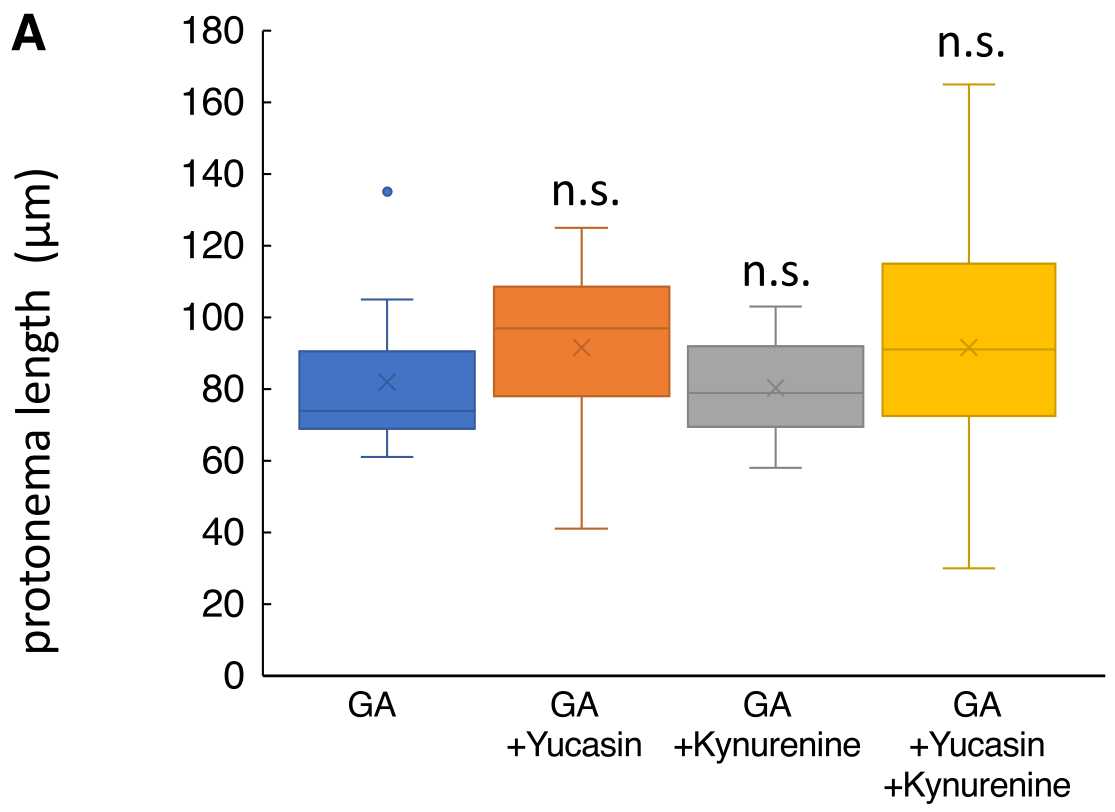
A
protonema length (μm)
180
160
140
120
100
80
60
40
20
0
n.s.
n.s.
n.s.
GA
GA +Yucasin
GA +Kynurenine
GA +Yucasin +Kynurenine

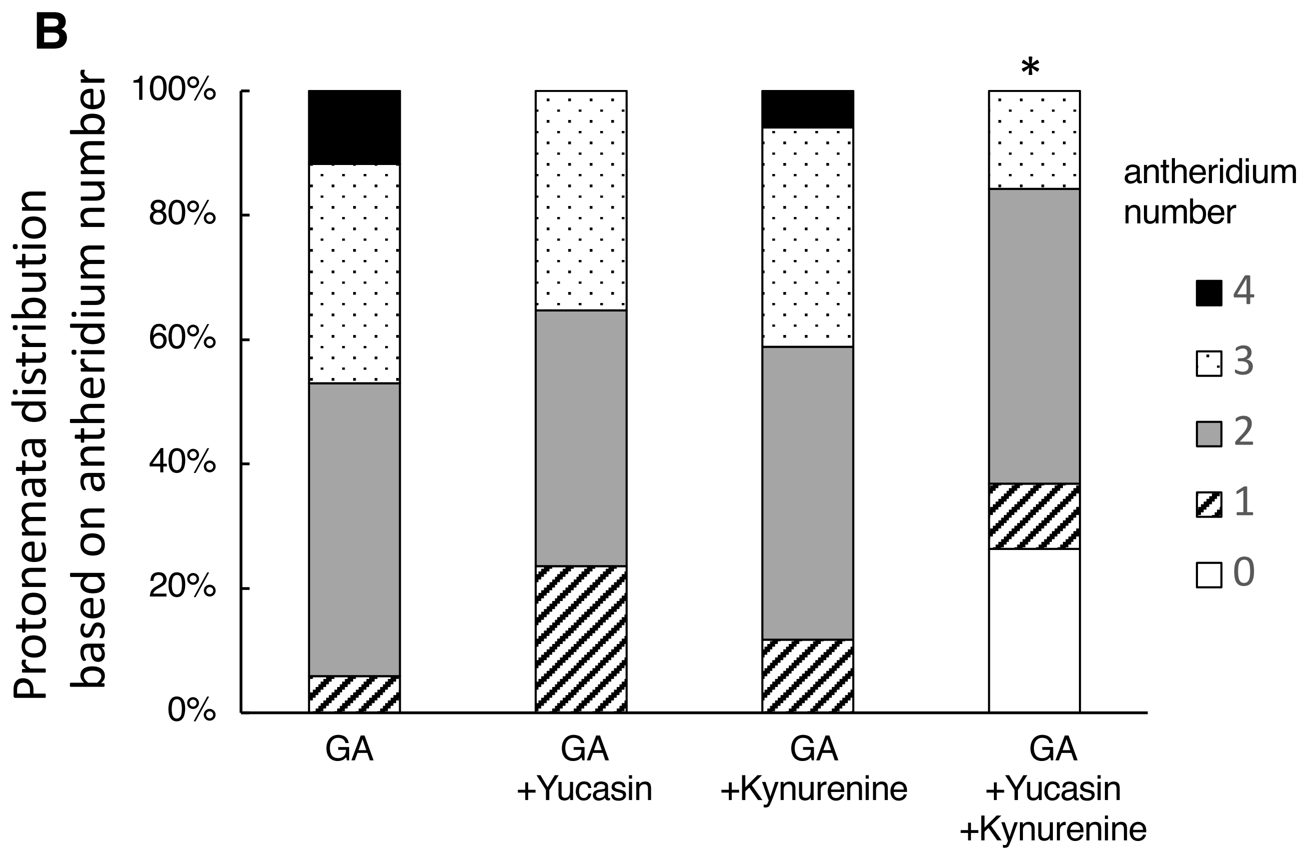
B
Protonemata distribution based on antheridium number
100%
80%
60%
40%
20%
0%
*
antheridium number
4
3
2
1
0
GA
GA +Yucasin
GA +Kynurenine
GA +Yucasin +Kynurenine

Figure S3

Table S1. Germination rate on medium containing GA_4-Me with TIBA.

TIBA concentration (μM)	0	1	10	50
Germination of spore (%)	38.4 (n=109)	32.2 (n=209)	39.1 (N=254)	5.0 (n=258)

Each MS medium contain 100 nM GA_4-Me.
Germination rate was measured at 7 days after red light irradiation to induce germination.
